# Supplementary material for: Are carbon emissions trading and green financial instruments synergistic? -Comprehensive quantitative research based on content analysis
Source: PLoS One. 2024 Mar 7;19(3):e0298601. doi: 10.1371/journal.pone.0298601 (PMC10919601; doi:10.1371/journal.pone.0298601)
Supplement: S1 Data — (ZIP) [file pone.0298601.s001.zip › Data/地级市Z_F工作报告.zip!/╣π╢1⁄2/│▒╓▌╩╨/└┤╫╘╣1⁄2╓┌║┼í╛┬φ┐╦╩2╛▌═°í┐_2007.docx]

各位代表：

我代表市人民政府向大会作工作报告，请予审议，并请政协各位委员和其他列席人员提出意见。

一、本届政府任期的工作回顾

2003年以来，在省委、省政府和市委的正确领导下，在市人大及其常委会和市政协的监督支持下，我们高举邓小平理论和“三个代表”重要思想伟大旗帜，以科学发展观统领政府工作全局，不断调整充实发展思路，团结广大干部群众开拓奋进，推动全市经济建设和社会各项事业较快发展，构建社会主义和谐社会取得显著成绩，较好地完成上次人代会提出的各项任务。

（一）经济发展逐年加快，经济实力明显增强

国民经济持续较快增长。四年来，全市经济增长速度都达到两位数，并呈现逐年加快趋势。2006年，预计实现生产总值331亿元，比上年增长13%；比2002年增加131亿元，年均递增11.8%；人均生产总值13090元，增长12.9%，年均递增11%。

产业结构进一步优化。经济结构战略性调整取得明显实效，第二产业的比重及工业中的重工业比重明显提高。三大产业结构从2002年的14.4:49.5:36.1调整优化为9.2:55.6:35.2。轻重工业的比例从80:20调整为65:35。

经济运行质量不断提高。财税收入保持较快增长。地方财政一般预算收入10.65亿元，比上年增长23.2%，比2002年增长一倍多，年均递增20.2%。加强非税收入管理取得明显成效。市本级实现非税收入5.28亿元，增长23.2%。各项税收总额33.31亿元（快报数），比2002年增长将近1.5倍。其中国税收入22.96亿元，地税收入10.35亿元，分别增长25%和22%，年均递增30.1%和20.3%。

金融存贷稳步增长。金融部门认真执行稳健货币政策，维护金融稳定。预计到去年底，全市金融机构本外币存款余额377.36亿元，各项贷款余额136.46亿元，分别增长14.7%和3.6%，年均递增16.8%和6.7%。

（二）特色产业快速发展，产业层次不断提高

抗灾复产取得全面胜利。去年，我市接二连三遭台风暴雨袭击，发生百年不遇的洪涝灾害，农业和渔业生产损失严重。在各级党委、政府的领导下，广大干部群众迅速掀起抗灾复产热潮，在最短的时间内恢复灾区的生产和生活秩序。社会各界和旅外乡亲捐款4500多万元，捐赠大米1325吨、衣服4.8万件等，为战胜洪涝灾害作出积极贡献。

落实支农惠农政策取得明显成效。加大投入力度，有效改善了农村的生产生活条件。去年，市级财政的涉农支出达5674万元，增长65.7%。义务教育阶段农村学生免收杂费，减轻农民负担5007.9万元。通过“三补贴”减轻农民负担652.6万元。安居工程实施三年来，各级政府已投入资金1667.5万元。四年来，农村公路建设投入资金5.4亿元。各项农村政策进一步落实，社会主义新农村建设扎实推进。

农业产业化有新进展。市级以上农业龙头企业25家，年销售收入34.5亿元，带动农户近7万户，户均增收1300多元。全市无公害农（渔）产品生产基地75个。水果、茶叶、蔬菜、花卉种植面积及效益不断提高。建立了柘林湾以网箱养殖为主的优质鱼类生产基地。人工鱼礁建设、伏季休渔等修复渔业资源工作取得新进展。预计农业总产值56.5亿元，下降8.6%。

工业生产和效益同步增长。预计工业总产值645亿元，其中规模以上工业总产值364亿元，分别增长15.1%和21%，年均递增11.7%和27%；工业经济效益指数136%，比上年提高2个百分点，比2002年提高45个百分点；利税总额21.5亿元，年均递增41%。

支柱行业主导作用明显。规模以上工业企业达800家，比2002年增加350家。其中年产值超亿元的企业有66家，超5亿元的6家，超10亿元的5家。规模以上七大行业实现增加值53亿元，占规模以上工业增加值的62.5%。陶瓷工业支柱作用突出，实现增加值24亿元，比重达到29%。

工业技术水平和创新能力明显提高。四年来，累计完成技改投资66.74亿元，新增生产线119条，新增生产设备8222台（套）。已被认定省级企业技术中心4家。累计实施国家、省、市各类科技计划项目598项，其中火炬计划项目101项。省级以上高新技术企业达101家，其中国家高新技术企业9家。预计高新技术产品产值100亿元，比2002年增长3.4倍。科技创新体系不断完善，企业自主创新能力跃居全省中上水平。在粤港关键领域重点突破项目招投标中连续五年中标。四年来共申请专利4195项，授权量2536项，居全省中上位置。

品牌带动战略取得丰硕成果。争创区域品牌成效突出，获得“中国优秀旅游城市”、“中国婚纱晚礼服名城”、“中国瓷都”、“中国潮州菜之乡”、“中国日用陶瓷特色产业基地”、“中国服装跨国采购产业基地”、广东省陶瓷、食品“产业集群升级示范区”等称号。还有4个专业镇获得区域性品牌称号。创建中国驰名商标和中国名牌产品实现零的突破。四年来，共获得中国驰名商标1件，中国名牌产品15项，国家免检免验产品23项，省著名商标49件，省名牌产品63项。累计获得省以上名牌名标155项。

第三产业稳步发展。文化旅游资源逐步转化为经济优势，古城文化旅游区建设扎实推进。建成开元寺大悲殿，动工修复广济桥、广济城楼、许驸马府、牌坊街等一批文物古迹。生态特色旅游初具规模，一批乡村生态旅游景点建成投用。预计接待海内外游客189.85万人次，旅游总收入27.48亿元，分别增长15.6%和15.4%，年均递增14.8%和14.6%。现代流通业、中介服务等第三产业全面发展。社会消费品零售总额117.4亿元，增长14%，年均递增12.2%。

（三）各项改革逐步深化，对外开放不断扩大

各项改革取得新的进展。初步建立起国有资产管理架构，国有企业改革不断深化。积极探索经营城市路子，推进投融资体制和市政公用事业建设体制改革。开展国库集中支付、部门预算、政府采购等改革，财政管理体制逐步完善。社会保障体系进一步健全，社会保险覆盖面逐年扩大。行政审批制度改革成效明显。教育、文化等方面的改革顺利推进。

开拓市场成效显著。先后举办了“中国瓷都——广东潮州陶瓷（北京）博览会”、“首届潮州国际陶瓷博览会”和“首届潮州文化旅游节”，组团赴深圳学习考察并举办潮州投资环境推介会，精心组织企业参加“广交会”、“首届广东国际服装博览会”，以及“泛珠三角”区域经贸合作洽谈会和中国国际中小企业博览会等，进一步拓宽国内外市场。

对外经济贸易持续快速增长。预计实现进出口总值23.18亿美元，增长22.6%，年均递增25.6%。其中出口总值17.9亿美元，增长21%，年均递增21.9%。陶瓷产品出口8.6亿美元，年均递增27.1%。机电产品和高新技术产品出口也大幅增长。全市出口额超过千万美元的企业达35家，比2002年增加17家。建成新的货柜车查验场，优化了通关环境。新签利用外资项目88个，比上年增加26个；实际利用外资0.69亿美元，增长16%。四年来实际利用外资3.07亿美元。

外事侨务工作有新突破。成功接待了泰国总理率领的访问团。与曼谷市实现互访并签署了缔结友好城市协议及旅游合作协议。组团赴澳门参加第十三届国际潮团联谊年会，并在年会期间举办了潮州陶瓷博览会、美食展示等活动，提高了知名度和美誉度。开展荣誉市民授荣活动，激发旅外乡亲的爱国爱乡热情。四年来共接受旅外乡亲捐资2.8亿元。对台经济、文化交流与合作有新进展。

（四）基础设施日臻完善，城市品位显著提高

固定资产投资力度明显增强。预计全社会固定资产投资总额110亿元，增长12.5%，年均递增29.5%。其中，更新改造30.8亿元，增长52.9%，年均递增30.1%。四年来固定资产投资累计332亿元，其中重点建设项目共148项，完成总投资105.88亿元。固定资产投资总额和重点建设项目的数量都创历史新高。

水利工程防灾减灾能力得到提高。全市列入省城乡水利防灾减灾工程项目14宗，总投资约6.1亿元。已动工11宗，累计完成投资3.06亿元。韩江东岸市区段防洪工程和秋西堤、东厢堤达标加固工程已完成。韩江潮州供水枢纽实现下闸蓄水。解决农村15.28万人饮水难工程、沿海6镇供水工程基本完成。

能源建设取得新突破。大唐三百门电厂首期2×60万千瓦燃煤机组建成投产。凤凰水电厂扩容改造、城市电网、农村电网改造等工程顺利完成。预计全市供电量39亿千瓦时，增长17.4%，年均递增19.6%。欧华能源成功在新加坡上市。与中海油总公司签订了能源战略合作协议，将在天然气储罐、城市管道燃气等方面进行合作。

交通建设成绩显著。完成全市干线公路网规划修编工作。争取厦深快速铁路在我市设立“两站一支线”工作顺利完成。四年来完成投资21亿元，新改建一级公路28.5公里，二级公路185.5公里，新改建县通镇公路62.8公里、镇通村公路1207.9公里。公路总里程达1939.2公里，每百平方公里公路密度62.9公里，分别比2002年增加66.1公里和2.1公里。港口新增吞吐能力240万吨。

城市品位显著提高。认真做好城市总体规划修编、专项规划和控制性详细规划编制等工作。加强规划监督管理，严肃查处违法违章建设行为。建设了人民广场、文化公园、厦寺公园、市第一污水处理厂二期工程、生活垃圾卫生填埋场、桥东水厂二期工程和汕汾高速公路延长线绿化工程。韩江北桥春节前将实现试通车。新建、改建了潮州大道、枫春路等一批城市主干道。四年来新增开放式活动场所约15万平方米，新增绿化覆盖面积121.39公顷。创建“国家园林城市”活动扎实推进，城市净化、绿化、美化、亮化水平不断提高，城市的吸引力和竞争力明显增强。城市管理长效工作机制初步形成，环境卫生、绿化养护的管理水平不断提高。潮安、饶平县城区及城镇建设也稳步推进。

社会信息化稳步发展。建设了全市统一的电子政务网络平台，促进了社会信息化建设。市电子政务专网、市宏观经济管理信息共享平台和潮州信用网等一批项目的应用取得成效。政府公众网在实现政务公开、便民服务、社会监督等方面发挥着积极的作用。教育、农业、山区、企业等方面的信息化建设迅速发展。社会信息化综合指数达到57.31。

（五）生态建设成效显著，持续发展得到保障

国土资源管理取得新成绩。坚决执行基本农田保护区“五个不准”规定，实现年度耕地占补平衡。四年累计补充耕地5122亩。认真开展土地市场治理整顿，清查处理各类违法用地行为。完成潮州供水枢纽、大唐电厂等重点建设项目的征地工作。办理“城中村”土地登记25742宗、161万平方米。矿产资源管理进一步加强，采矿权挂牌出让工作扎实推进。

环境保护与建设上新水平。扎实开展韩江、黄冈河、枫江流域环境和排污企业的综合整治，环境污染状况得到有效控制。建设项目全面落实环评制度，“三同时”执行率达到100%。市城区污水处理率达到60%，生活垃圾无害化处理率达100%。饮用水源水质、城市水域功能区、主要干流韩江水质保持在国家二级标准，近海功能区等水质达标率保持100%。连续六年在省环境保护责任考核中获优秀等次，去年是全省4个获优秀等次的城市之一。

林业建设与水土保持效果明显。加快以韩江流域水源涵养林、沿海防护林、生物防火林带为重点的林业建设，森林覆盖率达60.3%。创建了3个省级自然保护区和15个省级生态示范园（村）。韩江两岸已关闭采石场基本完成平面迹地的整治复绿，水土流失现象得到初步控制。

人口与计划生育工作得到加强。认真落实人口计生目标管理责任制，逐步完善利益导向机制，搞好计生优质服务。人口出生率、自然增长率分别从2002年的10.96‰、5.77‰下降到2006年的10.02‰、4.54‰，自然增长率连续8年控制在10‰以内，进入稳定低生育水平阶段。

（六）教育文化快速发展，各项事业不断进步

人才工作得到加强。人才市场信息化建设步伐加快。职称制度改革稳妥推进，专业技术资格评审管理逐步完善。出台了《潮州市工艺美术大师荣誉称号评审试行办法》等文件，工艺美术人才队伍建设得到加强。

教育水平进一步提高。“普九”成果得到巩固和提高。全市小学适龄儿童入学率为98.57%，初中入学率为95.61%，辍学率为1.99%，各项指标基本达到省的要求。累计投入资金5.74亿元，新建、扩建、改建学校350所，完成119所老区山区学校的改造，调整中小学校布局208所。市金山中学、高级中学和城南中英文学校新校址投入使用。优质教育资源快速增长，教学质量稳步提高。去年高考本科上线率首次高于全省平均水平。广东陶瓷职业技术学院已经动工。

文化事业蓬勃发展。文化设施建设力度加大，市文化艺术馆、博物馆等项目已动工，饶宗颐学术馆已经落成。文艺创作取得新成绩，40件作品在省级以上评比中获奖。送文化下乡、送戏下乡和广播电视“村村通”、“长期通”等工程取得良好效果。文化遗产保护工作得到加强，韩文公祠、从熙公祠和道韵楼被列为全国重点文物保护单位，潮州音乐、潮剧、潮绣艺术、潮州木雕、潮州铁枝木偶和潮州剪纸艺术被列入国家首批非物质文化遗产代表作名录。动漫制作、光盘生产等文化产业进一步发展。

国防建设、民兵预备役和双拥工作成效明显。连续第五次被授予“广东省双拥模范城”称号。国动委“十五”规划任务基本完成，民兵组织得到调整巩固，民兵和预备役人员在各项建设中发挥了积极的作用。

体育和其他各项事业取得新成绩。全民健身活动深入开展，体育竞技水平进一步提高。我市输送的运动员林跃在多哈亚运会跳水比赛中，勇夺男子十米台双人和单人两项冠军。全民健身广场建成开放，西湖山全民健身广场已动工。乡镇撤并任务顺利完成，基层民主建设得到加强。殡葬改革成果得到巩固发展，民间组织管理进一步规范。残疾人培训就业工作取得较好成绩。统计、民族、宗教、人防、地震、气象、档案和地方志等事业都取得新成绩。

精神文明建设进一步加强。“爱国、守法、诚信、知礼”现代公民道德建设取得新成效，以“八荣八耻”为主要内容的社会主义荣辱观教育深入开展。未成年人思想道德建设得到重视和加强。“四五”普法教育取得较好成果。群众性精神文明创建活动深入开展，城乡文明程度进一步提高。连续三届被评为“广东省文明城市”。

（七）人民生活水平不断提高，各项民心工程成效显著

就业形势保持稳定。全市累计新增就业岗位11.31万个，国有下岗失业人员实现再就业2.96万人，其中“4050”人员实现再就业7662人。累计再就业率达到76%。去年城镇登记失业率2.5%。

社会保险事业稳步推进。截至2006年底，全市参加养老、失业、工伤和医疗保险的人数分别为26.85万人、22.53万人、21.39万人和4.59万人，分别比2002年增长73.2%、74.1%、295.4%和200%。去年征收各项社保基金5.05亿元，累计结余4.82亿元，分别比2002年增长2倍多和3.2倍。

城乡居民收入增加。市区居民人均可支配收入9574元，农民人均纯收入4319元，分别增长7%和1.4%，年均递增7.2%和2.8%。居民消费水平稳中有升。市区居民人均消费性支出8733元，增长6.5%。

公共卫生事业有新发展。重新组建了市、县卫生监督所、疾病预防控制中心、卫生检验中心，建设“120”医疗急救指挥中心。处置非典、禽流感等突发公共卫生事件取得较好成效。参加新型农村合作医疗人数111.49万人，覆盖率达60%。卫生支农工作进一步加强，启动了“健康进农村”——潮州市巡回医疗安康工程。

解决“一保五难”工作扎实开展。列入最低生活保障2.19万户、4.94万人，占总人口的1.97%，基本做到应保尽保。农村饮水工程建设稳妥推进。各级挂钩扶贫工作深入开展，“十百千万”工程取得良好成效。已改造农村贫困户危房1450户。解决市城区“双困户”住房379户，是全省第三个提前完成解困任务的城市。县通镇、镇通村公路硬底化建设进展顺利。义务教育阶段农村学生免收杂费政策得到落实。

救灾救济工作有效推进。累计拨出救灾救济款6004万元，救助灾民100多万人次。去年因灾全倒户6090户住房重建已基本完成。慈善事业社会化有新发展。成立了潮州市儿童福利院和潮州慈善总会，慈善总会已接收捐款8000多万元。“春风暖人心”、“农村单亲特困母亲安居工程”、“同一片蓝天下”等扶贫济困工作取得显著成效。

（八）依法行政稳步推进，社会管理不断加强

民主法制建设取得新进展。坚决执行市人大及其常委会的决议，认真听取市人大代表、政协委员意见，加强与工会、共青团、妇联等群众团体的联系，认真听取人民群众对政府工作的意见和建议，落实重大问题集体决策制度、专家咨询制度、社会公示和听证制度，民主决策机制进一步完善。不断健全办理人大建议、政协提案工作网络。累计办理人大议案2件，办理代表建议294件，办理政协委员提案216件，办结满意率分别为98.6%和100%。制定规范性文件129宗。办理行政复议案件26宗。

依法行政工作全面推进。全面贯彻实施《行政许可法》和国务院《全面推进依法行政实施纲要》，行政许可实施机构减少为39个，行政许可事项减少为245项。落实行政执法责任制和执法过错责任追究制，强化对行政行为的监督制约。落实信访工作责任制，受理群众来信来访立案704宗，立案办结率100%。规范市长、县区长接访日和领导包案工作，解决了群众诉求的一批突出问题。

政府服务水平不断提高。扎实开展行政监察和机关效能建设，完善和落实机关内部的管理制度，推进政务公开，接受群众监督。开展为民服务全程办事代理制试点工作。成立了行政服务中心，累计受理业务4.14万件，办结率99.6%。认真履行六项廉政承诺，廉政建设责任制进一步落实。加强执法监察和专项检查，深入开展纠风专项治理和行风建设。审计监督工作得到加强。

社会治安保持稳定。健全矛盾纠纷排查调处机制，有效化解不安定因素。坚持“严打”方针，重拳打击严重暴力犯罪和“两抢一盗”等多发性犯罪活动，深入开展“打黑除恶”专项斗争，加大对“黄赌毒”、“六合彩”等违法犯罪活动的打击力度。进一步完善社会治安防控体系，启动“平安潮州”视频监控系统建设，强化对社会面的管理，及时消除各种治安隐患。

经济秩序继续好转。严厉打击侵犯知识产权、制假售假、非法传销、走私贩私、商业贿赂等违法犯罪行为，反对不正当竞争，营造公平、规范、有序的市场环境。食品安全信用体系建设取得阶段性成果，市政府被评为广东省食品安全先进单位。强化安全生产监管，开展专项整治，保持安全生产形势的基本稳定。

四年来的工作实践使我们体会到：一是必须坚持以经济建设为中心，不断加快经济发展步伐。要始终坚持把发展作为第一要务，大力发展特色经济，打响区域特色经济品牌，不断推动产业优化升级，促进潮州经济的较快发展。二是必须贯彻落实科学发展观，全面发展各项社会事业。要始终坚持全面发展、协调发展、安全发展、可持续发展，在加快经济发展的同时，努力改善发展环境，全面发展各项社会事业。三是必须切实关注民生，保障人民群众的基本权利。要始终坚持把实现好、维护好、发展好最广大人民的根本利益，作为一切工作的出发点和落脚点，认真解决关系民生的实际问题，让全体人民共享改革发展的成果。四是必须处理好改革与发展的关系，全力维护社会的稳定和谐。要始终坚持与时俱进，稳妥地推进各项改革，积极应对转型期出现的新情况、新问题，在加快发展的同时全力维护社会的安定团结。五是必须加强民主法制建设，提高依法行政水平。要始终坚持依法行政，规范行政行为，不断提高执政能力，做到科学行政、民主行政、依法行政。

各位代表，我市过去四年所取得的成就，是省委、省政府和市委正确领导的结果；是市人大及其常委会和市政协监督支持的结果；是全市人民贯彻落实邓小平理论、“三个代表”重要思想和科学发展观，辛勤劳动和奋力拼搏的结果；是驻潮部队、驻潮单位，各民主党派、人民团体及港澳台同胞、海外侨胞鼎力支持的结果。在此，我代表市人民政府表示衷心的感谢和崇高的敬意！

在肯定成绩的同时，我们也清醒地看到当前所面临的问题与困难：一是经济结构不够优化。工业经济总量不大，重化工业比重较小，产品附加值较低。二是生产要素制约经济发展。原材料价格上涨，石油气价格居高不下，影响工业的经济效益。信贷资金紧缺，严重制约工业技改和升级。工业用地供求矛盾突出，土地使用效益偏低，环境压力日益突出。三是农民增收难度较大。特别是去年受洪涝灾害影响，农业和渔业损失严重，影响农民增收的不利因素增多。四是市场秩序和经济管理仍存在很多薄弱环节，假冒伪劣、偷逃税费的现象时有发生，安全生产形势不容乐观。五是社会保障、文化建设等社会事业仍然滞后于经济发展。六是威胁群众生命财产安全的侵财型犯罪呈高发态势，各种利益矛盾突出，维护稳定的任务仍然比较艰巨。我们相信，新一届政府将继续采取有效措施解决存在问题，加快全市经济和社会的发展步伐。

二、今后五年的奋斗目标和2007年的工作建议

今后五年是我市经济和社会发展的重要时期。政府工作的指导思想是：以邓小平理论和“三个代表”重要思想为指导，以科学发展观统领经济和社会发展全局，认真贯彻落实省粤东工作会议和市第十二次党代会精神，坚持科学发展、优化结构、提高水平、全面推进的工作方针，突出“自主创新，品牌带动，市场开拓，港口建设，人才兴市”战略重点，做大做强特色经济、文化经济、港口经济，着力打造国内有重要影响力的历史文化名城、国内重要的特色产业基地和广东重要的能源石化基地，努力建设经济发达、文化繁荣、社会和谐、富裕安康的广东“东大门”，全面提升潮州改革开放和现代化建设水平。

今后五年，必须按照“三年打基础、五年大变化、十年大发展”的要求，全面加快经济和社会发展步伐，力争全市生产总值年均增长15%，达到全省平均水平；全市经济综合实力、城乡建设、社会发展和人民生活水平跃上新的台阶。要围绕这个目标，加快实施“十一五”规划，建设“一名城两基地”，推动全市经济又好又快发展。要积极调整优化产业结构，加强技术引进和自主创新，推动传统产业升级换代，积极开发建设潮州港经济区重化产业带，大力发展以历史文化名城为依托的特色文化旅游和第三产业，增强全市经济的发展后劲。要进一步巩固农业基础地位，大力发展县域经济，推进社会主义新农村建设。要加强基础设施建设，重视城乡环境保护。要重视区域经济合作，大力宣传推介优势产业、名牌产品和投资环境，积极开拓国内外市场，扩大招商引资成果，力争在吸引世界五百强企业方面有所突破。要坚持以人为本，更加关注民生问题，全面发展社会各项事业。要加强政府机关效能建设，进一步转变职能，提高依法行政水平。

必须多渠道筹措资金，实施一批关系全市经济和社会长远发展的重点项目，加快优化投资环境，增强城市综合竞争力和可持续发展能力。规划建设143个项目，规划总投资1468亿元。这些项目今后五年计划投资548亿元，其中：交通项目21项、113.9亿元；能源项目 18项、288.2亿元；水利项目16项、22.8亿元；工业项目31项、48.5亿元；农业项目3项、1.08亿元；服务业项目13项、17.5亿元；社会事业项目19项、17.7亿元；城建环保项目22项、38.4亿元。

今年是“三年打基础”重要的一年，国民经济和社会发展的预期目标是：生产总值增长14%（其中：第一产业增长4%，第二产业增长19%，第三产业增长12%）；单位生产总值能耗下降3.5%；地方财政一般预算收入增长20%；全社会固定资产投资增长20%；外贸出口总额增长16%；社会消费品零售总额增长15%；居民消费价格涨幅控制在2.5%；人口自然增长率控制在5.8‰以下；城镇登记失业率控制在3%以下；市区居民人均可支配收入增长7.5%；农村居民人均纯收入增长5%。

为实现上述目标，必须扎实抓好下列工作：

（一）调整优化产业结构

立足优势，打造特色产业基地。充分发挥“中国瓷都”和“中国婚纱晚礼服名城”等区域品牌的综合效应，大力培育一批具有自主品牌、核心竞争力强的大企业。整合现有工业园区，建设好市开发区二期和产业转移工业园，促进产业集聚发展。加快国家日用陶瓷特色产业基地建设，全力打造国内知名的陶瓷产品研发、制造、集散中心。加快国家日用陶瓷生产力促进中心、国家陶瓷产品质量监督中心和省食品质量安全监督检验中心筹建步伐。

加强规划建设，大力发展港口经济。充分利用潮州港的独特优势，严格实施潮州港经济区总体规划，多渠道增加资金投入，加强港口基础设施建设。积极配合加快梅潮高速公路建设，为临港工业发展创造良好条件。发挥大唐电厂、华丰石化基地等大项目的带动作用，抓紧开展大型炼油项目、液化天然气接收站和储备油基地的规划和招商，促进我市工业结构的调整。搞好港口服务设施配套，发展港口物流业、仓储业。

加快古城文化旅游区建设，发展特色文化旅游业。重新编制国家历史文化名城保护规划。加快广济桥二期、牌坊街、己略黄公祠等工程的施工进度。依托文物点创办具有潮州特色的专题展览馆。整合古城内外、一江两岸三桥主要景观，编制、策划古城文化旅游线路。大力整治古城区环境，完善古城区旅游功能配套。稳妥推进旧城区改造。搞好各类文化遗产的普查、保护和开发利用。保护和发展凤凰山畲族文化。加快发展特色文化旅游和乡村、工业旅游。进一步加强区域合作，加大力度宣传推介潮州特色旅游。

（二）切实加强农业和农村工作

巩固农业基础地位，加快产业化进程。抓好连片旱涝保收农田建设，提高农业综合生产能力。继续落实粮食工作考评制度，保护和建设好基本农田，努力提高粮食单产。大力发展优势特色产业，创建一批生柑无公害生产标准化示范区，加强潮州茶叶的品牌和质量标准建设，因地制宜发展“一乡一品”特色产业。继续扶持农业龙头企业和农产品流通大户，培育农民专业合作组织。完善15个类型农业生产基地的示范功能，建设好市级现代农业园区。积极开展农产品质量认证工作，培育名牌农产品。

调整优化渔业结构，促进捕捞、养殖协调发展。搞好渔港基础设施建设，改善渔业生产条件。建设深水抗风浪网箱养殖示范区，调整降低柘林湾内养殖密度。加大围垦区内养殖场地整治，推广无公害标准化养殖。完善渔业技术推广体系，发展水产品深加工，提高渔业生产效益。

加强社会主义新农村建设，推动农村经济社会全面进步。加大力度解决农村“一保五难”问题。进一步完善农村最低生活保障制度。继续抓好农村饮水工程、村村通公路和贫困户危房改造。加快农村改水、改厕、改路、改灶、改造住房步伐，建设一批生态文明村。实施农村基础教育“五项工程”，认真落实农村义务教育免收课本费的政策，确保今年底完成农村中小学校危房改造任务。加强农村文化设施建设。认真落实农村低保对象医疗救助制度，大力推进新型农村合作医疗工作，确保覆盖率达到80%以上。搞好农村财务管理、财务审计、会计代理制工作。继续做好扶贫工作，认真落实支农惠农政策，严肃查处坑农害农违法行为。

大力发展县域经济，增强整体经济实力。充分发挥资源优势，大力发展具有地方特色的工业龙头企业。加快工业园区建设，引导工业项目向园区集中，走新型工业化道路，增强县域经济发展后劲。进一步加强县城、中心镇和专业镇建设，加强镇、村规划工作，搞好城镇基础设施建设，增强城镇的辐射和带动功能。

（三）促进内外源经济协调发展

大力发展民营经济，进一步做大经济总量。建立健全有利于民营经济发展壮大的体制环境，扩大民营企业的投资经营领域，鼓励企业做大做强。引导企业开展股份制和股份合作制改造，帮助符合条件的企业改制上市，扩大生产规模。鼓励优势企业与国内外企业进行并购重组或技术合作，增强市场竞争力。

加强宣传推介，全方位开展招商引资。把招商引资工作放在全局性、战略性的位置，创新招商方式，拓宽招商渠道，扩大引资规模，提高利用外资的质量和水平。采取“走出去”和“引进来”相结合的办法，广泛宣传我市的产业、港口等优势和良好的投资环境。加强与侨团的联系沟通，争取以侨引商。要以企业为主体，园区为重点，开展产业招商、园区招商、港口招商和重点项目招商。鼓励现有外商投资企业增资扩股，支持企业通过转让经营权、出让股权等方式吸引外来投资。积极筹备办好第二届潮州文化旅游节。

发挥特色产业优势，提高对外贸易水平。引导企业创新产品，提高传统出口产品的附加值，扩大高新技术产品、机电产品出口。积极组织企业参加广交会，认真筹备2007年广州国际陶瓷展览会，有针对性地组织企业参加国际性大型会展，扩大对外贸易。在巩固传统出口市场的同时，大力拓展中东、拉美、非洲、俄罗斯、东盟等新市场。鼓励有条件的企业到境外创办公司，拓展国际贸易的空间。

加强区域经济合作，努力开拓国内市场。创造条件，主动承接珠三角地区的产业转移。加强与周边地区在资源开发、产业互补、交通对接、市场互通等方面的合作。积极组织企业参加国家和省举办的各种会展，加强与省内外重点区域的经济合作。

（四）增强自主创新能力

引导企业加强自主创新，不断完善科技创新体系。强化企业的自主创新主体地位，着力抓好高新技术企业、工业骨干企业、民营科技企业、农业龙头企业的自主创新工作。支持企业建立研发机构和技术中心，促进支柱产业的产学研结合。支持4家省级企业技术中心成为关键技术研发及产业化的主要载体，争取新增4家企业建立省级企业技术中心。新创办一批专业镇技术创新试点，加快民营科技园建设。建设好“广东省陶瓷研究创新开发平台”。引导社会资金和科技资源投入自主创新。

积极实施科技计划项目，促进科技成果及时转化。加快新技术、新材料、新设备的推广和运用，推广对改造传统产业有积极作用的科技成果。抓好去年结转和今年新开工的国债项目、“双高一优”项目、省结构调整重点项目的实施。鼓励引导符合条件的企业参与国家、省技术研发项目投标。积极申报国家和省2007年重点技术改造、技术创新项目。积极培育和发展技术市场。推动专利技术的实施和产业化。

加强企业信息化建设，加快新型工业化步伐。加快电子信息产品制造业和软件业发展。鼓励有条件的企业申报计算机系统集成资质认证。鼓励企业的信息化建设项目申报省市技改、科技项目。支持电子元器件、彩色数码显示器、发光二极管、只读和可录式光盘生产，引进开发集成电路设计和封装、光盘检测设备等项目。加强信息技术培训和研究开发，推动信息技术的普及。

推进实施品牌带动战略，增强产业核心竞争力。引导企业加强品牌建设，搞好自主品牌的宣传和保护。选定一批企业作为争创名牌名标重点单位予以扶持，争创国家名牌产品、驰名商标要有新的突破。扩大“中国瓷都”、“中国婚纱晚礼服名城”等区域性品牌的影响。鼓励和引导企业以名牌产品为龙头，通过各种合作形式，扩大生产经营规模。加强知识产权保护，营造有利于名牌成长的环境。

（五）大力推进基础设施建设

搞好水利工程建设，提高防灾减灾保障能力。加强水资源的统一规划和利用。认真做好列入省促进粤东发展计划9个水利项目的前期工作。抓好潮安内洋南总干排涝、沟尾溪排涝、湘桥区河内湖排涝3个涝区整治工程，加紧做好清淤清障和项目前期工作。继续实施城乡水利防灾减灾工程和农村饮水工程。抓紧实施小型水库除险加固、农村机电排灌设施改造、水库移民和小水电改造等项目。

加强交通、能源建设，改善投资环境。交通建设方面：积极配合做好厦深快速铁路潮州段建设。加快推进汕梅和潮揭高速公路潮州段施工，完成省道S231湘桥路段、省道S335潮州路段改造项目。潮州客运中心站、进港公路、韩江航道潮州段整治等项目要抓紧开工。能源建设方面：配合大唐电厂做好3、4号机组和风能发电项目前期工作，协调推进电厂配套电网建设。加快华丰石化基地二期工程建设进度。加紧做好中海油液化天然气、中远石油储备项目的前期工作，争取两大项目落户港区。

进一步完善城市基础设施，加快“创园”、“创模”进程。搞好城市总体规划修编和各项专项规划的编制。加强桥东片区的规划控制及基础设施建设，引导城市建设向东拓展。做好韩江南桥的选址及前期工作。动工建设绿榕南路，改造中山路、潮州大道北段和春荣路、新洋路，建设陈桥大排沟等城市排水系统，建设一批停车场、公共厕所和垃圾转运站，抓好管线下地工作。积极探索市政、环卫、园林设施的市场化管理新路子。普及“城中村”垃圾袋装化，完善市区全天候保洁制度。继续做好“国家园林城市”等创建工作，整治西湖公园，加快市区主要道路的绿化改造，提高城市美化、绿化、净化、亮化水平，为各项创建工作打好基础。加强城乡防震减灾工作，严格对建设工程地震安全性评价和抗震设防管理。妥善解决市城区房地产登记遗留问题。

（六）致力建设“生态潮州”

大力推进集约化生产，加快发展循环经济。加强资源综合利用，全面推进节能降耗，努力建设节约型社会。引导企业开展清洁生产，培育发展一批清洁生产企业。继续推进陶瓷废物和瓷土废沙回收利用等资源综合利用项目的实施。引导和帮助企业对现有陶瓷窑炉进行技术改造。构建企业、园区、城市和社会三个层面的循环经济框架，在资源开采、消耗、废弃物产生、再生资源产生和社会消费等方面，鼓励节约，抑制浪费。

重视生态保护和建设，增强可持续发展能力。在实施工业排污总量控制的基础上，全面实现达标排放，从源头上防治污染和保护生态。推进韩江环境整治，保护韩江、黄冈河等主要饮用水源。加快县城和中心镇污水和垃圾处理设施建设。严格落实新上项目环境影响评价制度，建立健全污染源监督管理系统。加强海洋生态环境保护，促进海洋资源的可持续利用。搞好生态功能区建设，开展生态绿色创建活动。继续抓好韩江两岸林分改造、生物防火林带等生态工程，提高森林覆盖率。

严格土地管理，加强矿产资源保护。认真落实耕地保护目标责任制，严肃查处违法用地行为。严格工业供地条件，提高单位土地投入强度，促进土地集约化利用。制订优惠措施，鼓励企业投资建设和使用标准厂房。认真实施市、县、镇土地利用总体规划，做好新一轮土地利用总体规划修编准备工作。深化矿业权改革，逐步建立矿业权市场。依法加强矿产资源管理，坚决查处乱采滥挖、破坏矿产资源行为。做好采石场关闭复绿、地质灾害防治等工作。加大海域综合开发和管理力度，优化海洋资源配置，制止利用无度、开发无序、使用无偿的用海行为。

（七）深化各项改革

完善公共财政体制。认真落实各项促收增收的措施，依法强化征管，做到应收尽收。深化“收支两条线”管理，清理和规范行政事业性收费。努力拓宽非税收入渠道，加强对政府资源占用收益收入、垄断性经营收入的征收管理。坚持集中财力办大事的理财方针，确保重点支出需要，并在力所能及的前提下增加对公共管理和公共服务性领域的投入。继续深化国库集中支付、部门预算、政府采购等改革。

提高金融服务水平。商业银行要以深化改革为动力，增加信贷资金供给，支持地方重点项目建设和支柱产业发展。深化农村信用社改革，增强服务“三农”的能力。积极创造条件，做好停业整顿的城市信用社和信托公司的市场退出工作。

深化国有企业改革。完善国有资产监管体系，切实加强国有资产监督管理。鼓励非公有制企业参与国有企业的产权制度改革，鼓励企业员工、经营者和社会投资者参股、控股，推进国有经济股份制改造，实现企业产权多元化。探索创新劣势企业退出机制，加快国有劣势企业依法、规范、有序退出市场步伐。

加快事业单位改革。深化事业单位分类改革。结合事业单位体制改革和人事制度改革，推进事业单位收入分配制度改革。加强事业单位机构编制管理。

继续整顿和规范市场经济秩序。加大物价监管力度，着力整治关系民生的价费突出问题。严厉打击制假售假、偷逃税费、无证无照经营等各类违法违规行为，进一步规范市场经济秩序。深入开展食品药品质量安全监管，抓好大案要案的查处工作。高度重视安全生产工作，层层落实安全生产责任制，建立健全监管体系。加强企业安全生产基础工作。深入开展专项整治，加大安全生产执法和责任追究力度，确保人民群众生命财产安全。

（八）促进社会和谐发展

推进教育全面均衡发展。合理配置教育资源，加大教育投入，提高中小学校办学水平。搞好农村教育标准化建设，推进城乡教育协调发展。继续发展高中阶段教育，推进规模化办学。重视发展职业技术教育，培养更多适应特色产业发展的技能型人才。抓好市职业技术学校与市农校的整合建设，加快市技工学校扩建工程进度。加大扶持和管理力度，促进民办职业技术教育健康发展。协助广东陶瓷职业技术学院加快建设进度。

搞好人才资源能力建设。加强人才市场建设，提高人才资源市场化配置服务水平。深化职称制度改革，探索建立科学的专业技术人才评价机制。根据产业升级优化的需要，切实抓好人才的引进培养工作。

积极发展文化和体育事业。健全公共文化服务体系，加强农村文化建设，扶持公益性文化事业发展。鼓励非公有资本进入政策许可的文化产业领域，培育和发展优势文化产业群。加强对重点文化项目、艺术团体的扶持引导，做好重要文化遗产和优秀民间艺术的保护和传承工作。广泛开展全民健身活动，增强市民体质。做好潮州市体育中心选址工作。

提高城乡医疗卫生保障水平。建立和完善公共卫生三大体系。加强疫情监测，防止传染性疾病的暴发流行。加强镇级疾控组织和计免规范化门诊建设。大力推进城市社区医疗卫生服务。积极推行廉价门诊、廉价病房、单病种限价收费，提高医疗收费的透明度。重视振兴中医药工作，扶持中医药事业发展。深入开展爱国卫生工作，提高城乡卫生水平。

稳定低生育水平。抓好各项避孕节育措施的落实，严格控制政策外出生特别是政策外多孩出生。完善人口计生利益导向机制。健全流动人口计生管理服务机制。大力治理出生性别比失衡问题。加强计生分类指导，争取计生工作达到全省中上水平。

加强就业和再就业工作。积极推进城乡统筹就业，大力发展非公有制经济和第三产业，特别是社区服务业、劳动密集型产业，创造更多的就业机会。继续推进小额担保贷款、社保补贴、岗位补贴、公益性岗位开发工作，重点帮扶“4050”人员以及“零就业家庭”人员就业。加强职业技能培训，促进农村富余劳动力转移就业。认真组织实施好退役士兵免费职业技能培训工作。

健全多层次的社会保障体系。推进建立社会保险、社会救助、社会福利、慈善事业相结合，基本覆盖城乡居民的社会保障体系。大力推进社会保险扩面征缴工作。完善职工医疗保险办法，启动生育保险，制订被征地农民养老保险办法。完善社会救助体系，发展慈善事业和残疾人事业，为城乡困难群众排忧解难。增加经济适用房和廉租房供给。

重视国防动员建设。加强国防和民兵预备役工作，深入开展双拥共建活动，积极争创全国双拥模范城，促进经济与国防建设协调发展。

全力维护社会稳定。建立健全公共突发事件预警和应急工作机制，提高处置公共突发事件的能力。加强信访工作，及时发现和化解社会矛盾。建立健全矛盾纠纷排查调处机制和应急处置机制，积极预防和妥善处置群体性事件。保持“严打”高压态势，完善治安防控体系，推进和谐社区建设，切实维护社会稳定。

（九）推进精神文明和民主法制建设

加强精神文明建设。大力弘扬民族精神和时代精神，加强公民思想道德体系建设。扎实推行“五五普法”活动。重视青少年思想道德建设，完善未成年人学校、家庭、社会三位一体的教育网络。深入开展群众性精神文明创建活动，全面提升城乡文明程度。

发展民主政治。坚持向人大及其常委会报告工作、向人民政协通报情况的制度，自觉接受监督。认真办理人大议案、代表建议和政协提案。发挥民主党派、工商联、无党派人士参政议政作用和工会、共青团、妇联组织的桥梁纽带作用。扩大基层民主，完善村民自治、居民自治等基层民主管理制度。

全面推进依法行政。认真贯彻落实国务院《全面推进依法行政实施纲要》，努力建设法治政府，提高政府规范性文件的质量。深入实施《行政许可法》，深化行政审批制度改革，规范审批程序。积极推进“网上审批”和“一站式”电子政务。全面推行行政执法责任制，加强行政执法监督，促进严格执法、公正执法、文明执法。进一步提高审计监督工作水平。

大力推行政务公开。依法扩大政务公开的范围，规范公开内容，丰富公开形式。运用行政权力办理的与人民群众利益相关的各类事项，要依法、及时向社会公开。积极推进电子政务建设，提高政府工作透明度和公众参与度，自觉接受新闻舆论和人民群众的监督。

切实加强政风建设。深化机关效能建设，推广为民服务全程代理制，提高行政机关办事效率。落实党风廉政建设责任制，强化监督检查和责任追究，加强执法监察，严肃查办违纪违规行为，重点抓好治理商业贿赂工作，纠正损害群众利益的不正之风，加大力度从源头上预防和治理腐败。严格执行廉洁自律各项规定，扎实推进政风行风建设。

各位代表，今年是我们贯彻落实省粤东工作会议和市第十二次党代会精神，加快经济和社会发展重要的一年。我们要紧密地团结在以胡锦涛同志为总书记的党中央周围，高举邓小平理论和“三个代表”重要思想伟大旗帜，认真贯彻落实科学发展观，在市委的领导下，团结拼搏，开拓创新，为夺取潮州现代化建设的新胜利而努力奋斗！
